# Key performance indicators for monitoring of anemia management and iron status in children attending a pediatric dialysis unit: the experience of Ain Shams University

**DOI:** 10.1007/s40620-024-02017-3

**Published:** 2024-07-18

**Authors:** Mariam A. Khalil, Mohammed F. Kasem, Ihab Z. El-Hakim, Amany M. Abd Elhafez, Mohamed S. El Farsy, Ragia M. Said

**Affiliations:** 1 Division of Pediatric Nephrology, Department of Pediatrics, Faculty of Medicine, Ain Shams University, Cairo, Egypt; 2 Department of Community, Faculty of Medicine, Ain Shams University, Cairo, Egypt

**Keywords:** Key performance indicators (KPIs), Hemodialysis (HD), Chronic kidney disease (CKD), Anemia of CKD

## Abstract

**Background:**

Anemia in children on maintenance hemodialysis (HD) leads to poor quality of life. Our study aimed to assess and monitor anemia and iron status management in children on maintenance HD over 18 months using key performance indicators.

**Methods:**

Key performance indicators, formulated as the percentage of patients achieving the KDIGO (2012) guideline-recommended targets for hemoglobin (Hb) (11–12 g/dl), transferrin saturation (TSAT) (20–40%) and serum ferritin (200–500 ng/ml), were reported quarterly over the 18-month-period of this study.

**Results:**

This study was carried out over an 18 month-period, from April 1st, 2020, till October 31st, 2021. A total of 78 patients (45 males and 33 females) were included; mean age 12.16 ± 3.3 years and HD duration range 3.0—140.88 months, median 16.51 months. The three most common primary causes of CKD were Congenital Anomalies of the Kidney and Urinary Tract (CAKUT) (29.5%), unknown cause (24.4%), and chronic glomerular diseases (20.5%). The quarterly reported percentages of patients achieving the recommended targets for Hb, TSAT, and serum ferritin ranges were 18.2–35.7%, 38.8–57.1%, and 11.9–26.6%, respectively.

**Conclusion:**

Although the mean Hb trend was nearing the KDIGO (2012) target, the key performance indicators showed that only a small percentage of our HD patients were achieving the targets for Hb, TSAT, & serum ferritin, thus alerting us to the need to revise our protocol for the management of anemia and iron status.

**Graphical abstract:**

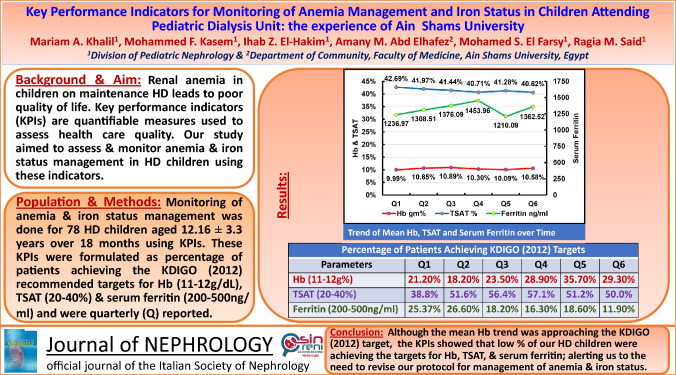

## Introduction

Anemia is a challenging complication in children on maintenance hemodialysis (HD) as its presence affects their physical, cognitive, and mental fitness. When severe, anemia is associated with cardiovascular dysfunction, cardiomyopathy, and death.

Key performance indicators (KPIs) are quantifiable measures of overall performance over a specific period for a specific objective [1]*.* Key performance indicators for anemia management and iron status in CKD patients on maintenance HD are calculated as the percentage of patients achieving the KDIGO (2012) recommended targets for Hb concentration, TSAT, and serum ferritin. These key performance indicators reflect how far HD units can ensure and monitor proper management of anemia and iron status delivered to their patients [2].

This study aimed to assess and monitor anemia and iron status management in children on maintenance HD attending the Pediatric Dialysis Unit at Children’s Hospital of Ain Shams University over an 18-month period using key performance indicators for Hb, TSAT, and serum ferritin.

## Subjects and methods

This study was conducted at the Pediatric Dialysis Unit, Children’s Hospital of Ain Shams University over an 18-month period from April 1st, 2020, till October 31st, 2021. The study was approved by the Research Ethics Committee, Faculty of Medicine, Ain Shams University (FWA 000017585; FMASU: MD 227/2020) and was performed according to the guidelines and Declaration of Helsinki. Seventy-eight incident CKD patients aged 18 years or less on thrice-weekly maintenance HD for at least 3 months were recruited.

Data extraction sheet included patients’ demographic information, cause of CKD, detailed HD information, parameters for anemia including hemoglobin (Hb) concentration, transferrin saturation (TSAT), and serum ferritin, administered erythropoiesis-stimulating agents (ESAs) and intravenous iron therapy, spKt/V, serum albumin, and parathyroid hormone (PTH). All patients were receiving ESAs, whether epoetin alfa 150–300 IU/kg/week or darbepoetin alfa 0.45 mcg/kg/week, and intravenous iron therapy as per our anemia management protocol. The doses of ESAs and iron therapy were continuously modified according to the results of Hb concentration, TSAT, and serum ferritin. As per KDIGO (2012) guidelines [3], Hb concentration was measured monthly, while TSAT and serum ferritin levels were measured every 3 months targeting a Hb concentration of 11–12 g/dl, TSAT 20–40%, and serum ferritin level 200–500 ng/ml. At the end of each quarter, the key performance indicators were calculated as the percentage of patients that achieved these targets. Absolute iron deficiency (AID) anemia was defined as anemia associated with TSAT < 20% and with serum ferritin < 200ng/ml, while functional iron deficiency (FID) anemia was defined as anemia associated with TSAT ≤ 20% and serum ferritin > 200ng/ml [4].

Data were analyzed on an IBM personal computer, using Statistical Program for Social Science version 26, (SPSS Inc., and Chicago, IL, USA). Descriptive statistics were used, and data were expressed as percentage, mean ± standard deviation (*x̄* ± SD) or median, and interquartile range (IQR). The Shapiro–Wilk test for normality was used. Frequency distributions were compared using Pearson’s Chi-square test and Fisher’s exact test. Phi and Cramér’s V were used to measure the strength of association between categorical parameters. To study correlations, we used Pearson correlation and Spearman’s rank correlation tests. *p* value was considered significant if less than 0.05.

## Results

A total of 78 patients were enrolled in this study with a mean age of 12.16 ± 3.3 (3.6–17.6) years. There were 45 (57.7%) males and 33 (42.3%) females. The three most common primary causes of CKD were Congenital Anomalies of the Kidney and Urinary Tract (CAKUT) in 29.5%, unknown cause in 24.4%, and chronic glomerular disease in 20.5% of our patients.

All patients were receiving 3-h HD sessions every other day (3 sessions/week). Hemodialysis duration range was 3.0–140.88 (median = 16.51 and IQR = 39.7) months. Twenty-four patients were on conventional HD (30.8%), 13 patients (16.7%) were exclusively on online hemodiafiltration (OL-HDF), 29 patients (37.2%) were receiving 2 of the thrice weekly sessions as OL-HDF, and 12 patients (15.3%) were receiving 1 of the thrice weekly sessions as OL-HDF.

The weekly prescribed dose of ESAs was 205.85 ± 43.47 IU/Kg of intravenous Epoetin alfa in 68 (87.2%) patients and 0.6 ± 0.15mcg/Kg of intravenous Darbepoetin alfa in 10 (12.8%) patients. We did not find any significant differences in ESA dose in any of the 6 quarters of the study between anemic and non-anemic patients (*p* > 0.05).

**Fig. 1:**
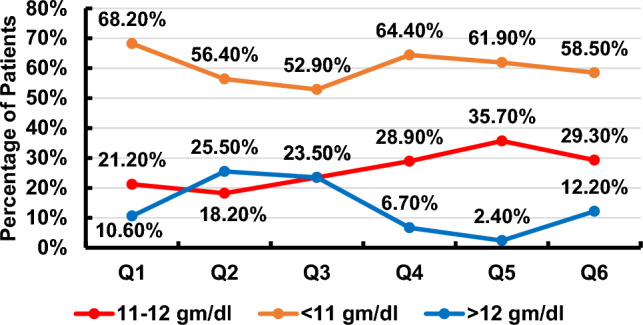
Key performance indicator for hemoglobin concentration (Q: quarter)

**Fig. 2:**
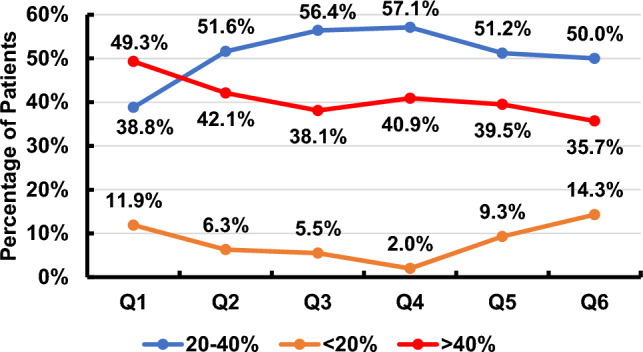
Key performance indicator for transferrin saturation % (TSAT%) (Q: quarter)

**Fig. 3:**
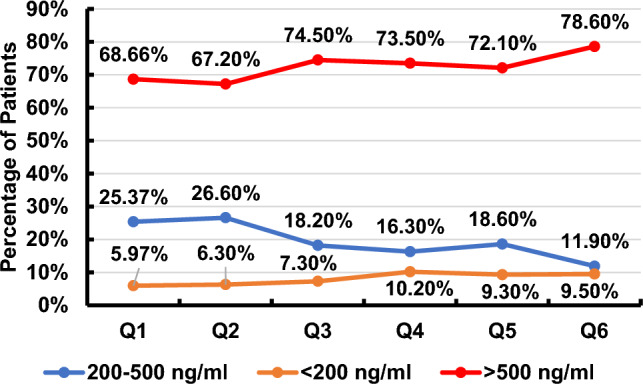
Key performance indicators for serum ferritin level (Q: quarter)

**Fig. 4:**
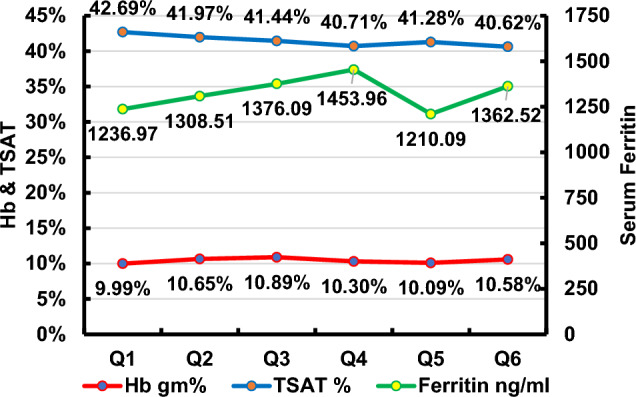
Trend of mean Hb, TSAT and serum ferritin over time

The quarterly reported key performance indicators for Hb, TSAT, and serum ferritin are illustrated in Figs. 1, 2, 3. The recommended targets for Hb, TSAT, and serum ferritin were achieved in 18.2–35.7%, 16.3–29.7%, and 11.9–26.6% of patients, respectively, throughout the 6 quarters of the study. The percentages of patients with readings below the targets for the 3 parameters were 52.9–68.2% for Hb, 2.0–14.3% for TSAT and 5.97–10.2% for serum ferritin throughout the 6 quarters of the study. The percentages of patients with readings above the targets for the same parameters were 2.4–25.5% for Hb, 64.1–74.4% for TSAT, and 67.2–78.6% for serum ferritin throughout the 6 quarters of the study. The trend of the mean value of each of the three variables over time is illustrated in Fig. 4***.*** There was statistical evidence of a significant increase in mean Hb when comparing its value in the 1st quarter to each one of the subsequent five quarters (*p* = 0.002, *p* = 0.002, *p* = 0.04, *p* = 0.023, *p* = 0.03, respectively). There was no association between the presence of anemia, Hb, TSAT, or serum ferritin with spKt/V at any of the study time points.

Parathyroid hormone levels were higher in anemic versus non-anemic patients in the 2nd (*p* = 0.003), 4th (*p* = 0.002) and 6th (*p* = 0.003) quarters and were comparable in the 1st, 3rd, and 5th quarters (*p* > 0.05). There was no association between the presence of anemia and hyperparathyroidism (PTH level > 300 pg/dL) (*p* > 0.05). Similarly, there was no association between the presence of anemia and each of age, sex, cause of CKD, HD modality, and serum albumin (*p* > 0.05). There were significant associations between the presence of anemia in our patients at the time of enrollment in the study and both HD durations. i.e., < 6 months (*p* < 0.05) and < 12 months (*p* < 0.05). There was no correlation between Hb and serum ferritin at any time point of the study.

The study started with 78 patients but ended with 42 patients. Throughout the 18 months of the study, 22 (28.2%) patients transferred—mainly to an adult service, 8 (10.3%) underwent renal transplantation, and 6 (7.7%) died, namely from bronchopneumonia and COVID-19 infection.

## Discussion

All our patients had been on ESAs and intravenous iron therapy since HD initiation and they had to have been on HD for at least 3 months as a prerequisite to be included in the study. Despite this, 59.7% of them had Hb < 11g/dl at the time of enrollment. Throughout the study this percentage fluctuated between 52.9% and 68.2%. Our data are comparable to the values reported by the 2001 North American Pediatric Renal Trials and Cooperative Studies (NAPRTCS) [5, 6], and higher compared to values reported by Frankenfield et al. [7] and Fadrowski et al. [8]. On the other hand, 99% of Sudanese children on HD were anemic despite extensive use of rHuEPO and iron supplementation [9].

We did not find any association between the presence of anemia and each of age, sex, and cause of CKD. Abdelmageed et al. [9] reported similar results. Fadrowski et al. [8] found the same results except that they showed an increase in anemia as age increased. There was significant association between the presence of anemia in our patients at the time of enrollment into the study and HD duration < 6 months. Abdelmageed et al. [9] and Fadrowski et al. [8] reported the same finding. We did not find any association between the presence of anemia and either spKt/V or any of the HD modalities. Fadrowski et al. [8] and Smith et al. [10] reported the same regarding spKt/V and anemia.

Serum ferritin and TSAT are the most commonly used measurements to determine iron status in patients on regular HD. Absolute iron deficiency anemia was found in 1.8–4.5% of our patients. Abdelmageed et al. [9] reported absolute iron deficiency in 4.17% of their patients. Like ours, Fadrowski et al. [8] did not show any significant association between the presence of anemia and TSAT or serum ferritin. According to Gaweda [11], due to biological and analytic variability of TSAT and serum ferritin, single measurements had limited diagnostic value in evaluating iron status in HD patients and they should be measured at quarterly intervals, just as we did in our study.

One of the reasons for rhEPO-resistant anemia in CKD patients might be attributed to functional iron deficiency associated with impaired iron availability [12]. In our cohort, however, only 2.4–4.2% of anemic patients fulfilled the criteria for functional iron deficiency. In HD patients, functional iron deficiency anemia could be attributed to suboptimal HD doses, ESA therapy, malnutrition-inflammation complex syndrome, and hyperparathyroidism.

We did not find any association between spKt/V and the presence of anemia or the changes in Hb, TSAT & serum ferritin. Nonetheless, the probability of suboptimal HD is still to be considered as all of our patients were receiving 9 HD-hours/week.

Erythropoiesis-stimulating agent therapy causes the bone marrow to strip iron off the circulating transferrin faster than it can replenish it from the iron stores [12]. We found no significant differences in ESA dose between anemic and non-anemic patients, nor any significant correlations between Hb levels and ESA doses, yet the calculated mean doses of ESAs might still be low. This issue is mainly related to financial and drug availability problems.

Like ours, nearly all the studies carried out on anemia in HD patients failed to find any significant association between the prevalence and severity of anemia and secondary hyperparathyroidism. All evidence of the role of PTH in renal anemia is indirect and based on the observation that parathyroidectomy in CKD patients is often followed by a rise in Hb level and a decrease in ESA dose [10].

Renal Association clinical practice guideline (2017) recommended Hb monitoring be performed prior to a mid-week HD session in HD patients receiving thrice weekly dialysis as this would minimize Hb variability due to over-hydration secondary to the longer inter-dialytic interval [13]. Unfortunately, sampling of our patients was not established prior to a mid-week HD session.

There are no available data addressing the treatment of anemic HD pediatric patients with low TSAT but high serum ferritin. We need to make sure that pediatric patients in this condition are administered optimum HD with ultrapure dialysis water, receive treatment for secondary hyperparathyroidism, are ensured proper nutrition with careful attention to micronutrients, and that other causes of anemia, especially missed hereditary conditions like β Thalassemia Minor, are excluded. We should also consider that serum ferritin represents only 1% of the total iron pool and behaves as an acute-phase reactant. The use of intravenous iron in anemic HD children with low TSAT but high ferritin needs to be studied in multicenter randomized controlled trials to assess its efficacy and safety. In the meantime, we can cautiously use intravenous iron on an individualized basis, weighing the risk–benefit and aiming to achieve TSAT of no more than 50% and a serum ferritin level no higher than 1000 ng/ml. In this case serum ferritin and TSAT assessment should be carried out every month. Extrapolating from the clinical practice guidelines used to evaluate iron overload in children with β-Thalassemia Major, assessment of hepatic and / or cardiac iron using MRI might be considered if serum ferritin is ≥ 1000 ng/ml [14]. Unfortunately, sampling of our patients was not established prior to a mid-week HD session and as such must be considered a limitation of the study. However, the results of this study give us a reason to conduct some research using more sensitive tools for monitoring anemia and iron status in such patients, like reticulocyte Hb content, percentage of hypochromic RBCs, soluble transferrin receptors, hepcidin, and hypoxia-inducible factor.

## Conclusion

To our knowledge, this is the first study using the percentage of patients achieving the benchmark targets of Hb, TSAT, and serum ferritin as key performance indicators for continuous monitoring of anemia and iron status management in children on maintenance HD. Although the mean Hb trend was close to the KDIGO (2012) target, the key performance indicators showed that a small percentage of our HD children were achieving the targets for Hb, TSAT, & serum ferritin; alerting us to the need to revise our protocol for the management of anemia & iron status.

Despite these results, there is still great potential for improvement in the attainment of these key performance indicators. We need to dig deeper to identify additive causes in the pathogenesis of anemia in our patients, searching for immunologic causes, like in SLE and HUS, antiESA-antibodies, trace elements and vitamin deficiencies.  Review of concomitant medications, search for  hereditary anemias, identifying chronic heavy metal intoxication, and hidden blood loss are further important steps. Internal and external auditing should be followed by a revision of our clinical practice and staff training.

## Data Availability

Data will be available upon request to the corresponding author.
